# Diffuse large B‐cell lymphoma arising immediately after completion of treatment for Hodgkin lymphoma

**DOI:** 10.1002/jha2.295

**Published:** 2021-09-21

**Authors:** Ai Hirota, Masahiro Yokoyama, Norihito Inoue, Yuko Shirouchi, Kengo Takeuchi, Dai Maruyama

**Affiliations:** ^1^ Department of Hematology Oncology Japanese Foundation for Cancer Research Cancer Institute Hospital Tokyo Japan; ^2^ Division of Pathology Japanese Foundation for Cancer Research Cancer Institute Tokyo Japan; ^3^ Department of Pathology Japanese Foundation for Cancer Research Cancer Institute Hospital Tokyo Japan; ^4^ Pathology Project for Molecular Targets Cancer Institute Japanese Foundation for Cancer Research Tokyo Japan

**FIGURE 1:**
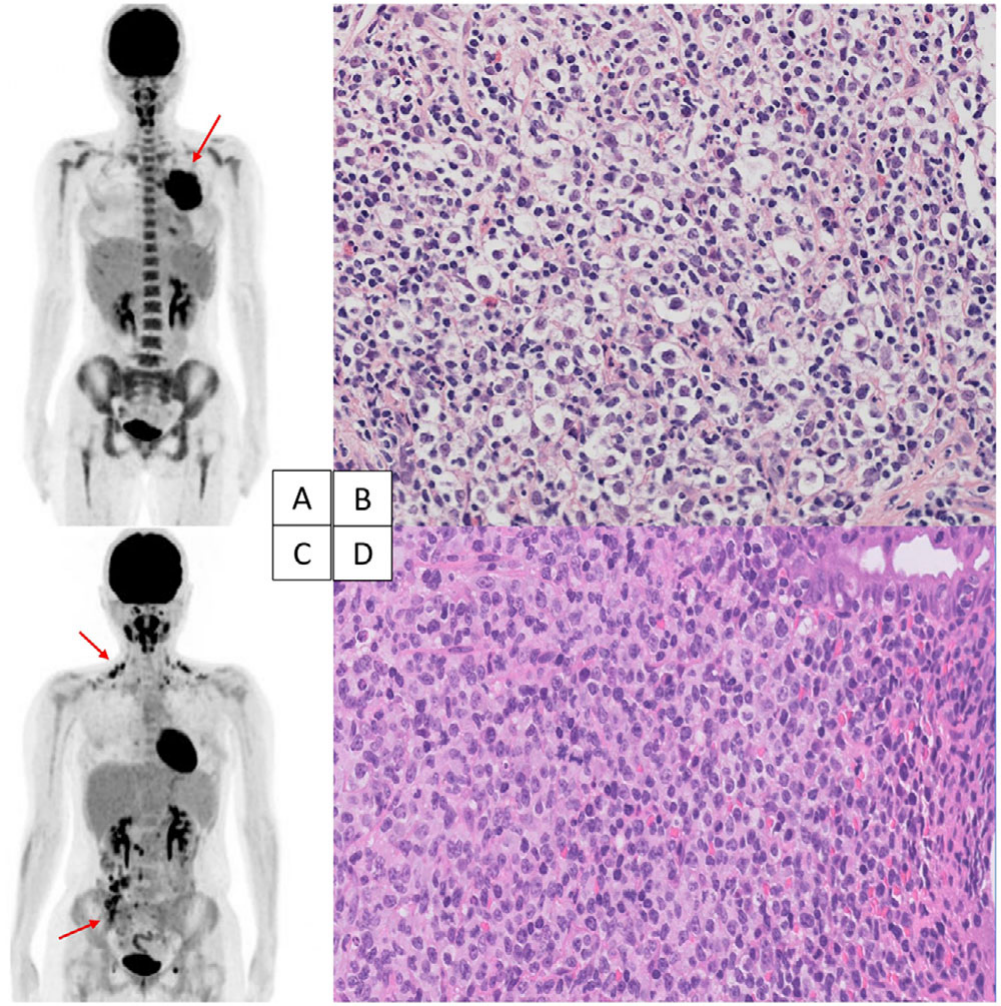
FDG‐PET/CT and histopathological findings: nodular sclerosis classic Hodgkin lymphoma (A and B), diffuse large B‐cell lymphoma (C and D).

A 19‐year‐old woman was referred to our hospital from a local clinic for evaluation of a mediastinal mass. ^18^F‐fluorodeoxyglucose‐positron emission tomography/computed tomography (FDG‐PET/CT) showed increased FDG uptake in a bulky anterior mediastinal tumor (SUVmax, 21.8) and multiple mediastinal and left hilar lymph nodes (Figure 1A). Histopathological examination of the needle biopsy specimen from the mediastinal mass showed nodules surrounded by fibrous collagen bands. In the nodules, Hodgkin/Reed‐Sternberg (HRS) cells were sparsely distributed in the background of various inflammatory cells (Figure 1B; objective lens, 400×). Immunohistochemistry demonstrated that the HRS cells were CD20^–^, CD30^+^, CD15 weak focally^+^, PAX5^+^, and EBER^–^. The patient was diagnosed with nodular sclerosis classic Hodgkin lymphoma (NSCHL), stage IIA, and underwent six cycles of brentuximab vedotin (BV) with doxorubicin, vinblastine, and dacarbazine (AVD) chemotherapy.

Although FDG‐PET/CT performed at the end of treatment showed complete regression of the initial lesions, new bilateral cervical, mesenteric, and ileocecal lymph node lesions with FDG uptake appeared (SUVmax, 8.3; Figure 1C). Histopathological examination of the ileum biopsy specimen by colonoscopy identified diffuse proliferation of large lymphoid cells in the lamina propria (Figure 1D; objective lens, 400×), and immunohistochemistry of the tumor cells showed CD10^–^, CD20^+^, CD30^+^, CD15^–^, and EBER^–^ cells. The patient was diagnosed with diffuse large B‐cell lymphoma (DLBCL) and was then treated with rituximab‐containing chemotherapy, achieving a complete metabolic response.

Progression of the new lesions to NSCHL was initially considered, but because the clinical course and distribution of the lesions were atypical, re‐biopsy was performed. Although no DLBCL was found in the pathological specimen at the time of initial presentation, it is possible that DLBCL was present in areas that were not biopsied or that the lesions changed to DLBCL during treatment. When a patient with CHL displays disease progression, especially at different sites, immediately after standard therapy such as BV‐AVD and displays an uncommon clinical course, the possibility of DLBCL arising should be considered.

When a patient with CHL progress immediately, especially in different sites, after standard therapy such as BV‐AVD and displays an uncommon clinical course, it is necessary to consider the possibility of other lymphoma subtypes, such as DLBCL, arising.

